# Trophic indices for micronektonic fishes reveal their dependence on the microbial system in the North Atlantic

**DOI:** 10.1038/s41598-021-87767-x

**Published:** 2021-04-19

**Authors:** Antonio Bode, M. Pilar Olivar, Santiago Hernández-León

**Affiliations:** 1 Centro Oceanográfico de A Coruña, Instituto Español de Oceanografía, IEO, 15080 A Coruña, Spain; 2 Institut de Ciències del Mar, CSIC, 08003 Barcelona, Spain; 3 Unidad Asociada ULPGC-CSIC, Instituto de Oceanografía y Cambio Global, IOCAG, Universidad de Las Palmas de Gran Canaria, Campus de Taliarte, 35214 Telde, Gran Canaria Spain

**Keywords:** Stable isotope analysis, Marine biology

## Abstract

The importance of microbes for the functioning of oceanic food webs is well established, but their relevance for top consumers is still poorly appreciated. Large differences in individual size, and consequently in growth rates and the relevant spatial and temporal scales involved, make the integration of microorganisms and large metazoans in a common food web framework difficult. Using stable isotopes, this study estimated the trophic position of 13 species of micronektonic fishes to examine the microbial and metazoan contribution to mid trophic level consumers. Vertically migrant species displayed higher trophic positions than non-migrant species in all depth layers. The estimated trophic positions agreed well with those from the literature, but all species displayed mean increases between 0.5 and 0.8 trophic positions when taking into account microbial trophic steps. Trophic position, but not the relative importance of the microbial food web, increased with individual size, suggesting that current estimates of the trophic position of top consumers and of the length of oceanic food webs are too low because they are based only on metazoan trophic steps. This finding calls for a review of trophic position estimates and of the efficiency of trophic transfers along oceanic food webs.

## Introduction

Since the discovery of major trophic pathways in the microbial system of the ocean four decades ago^1^ their full integration in the whole food web remained elusive. The current paradigm considers oceanic food webs organized in a continuum of cases between two main types of trophic systems: microbial and metazoan food webs^2,3^. The former is mainly related to remineralisation and respiration^3^ but also to the sequestration of carbon in the form of recalcitrant dissolved organic compounds^4,5^. In contrast, the efficient transfer of organic matter to upper trophic levels (e.g. fisheries^6^), the sequestration of carbon in the deep ocean by sedimenting particles^7^ and the active flux by zooplankton^8,9^ are mainly attributed to the metazoan food web^10,11^. Both trophic systems, however, are connected by the protozoan grazers allowing large-sized metazoans to consume the otherwise inaccessible dissolved organic matter concentrated by bacteria.

The study of the links between both trophic systems in the ocean has been limited by the large difference in the time and space scales relevant for microbes and large animals^12^. Diet studies are unable to capture all the complexity of the food web because of the difficulties in identifying all prey items^13^. Integrated approaches using tracers (e.g. stable isotopes) are limited by the ‘invisibility’ of microbial pathways to traditional models for estimating trophic positions^14^. Consequently, most food web models consider mainly metazoan links and therefore underestimate true trophic positions of top consumers, as evidenced by meta-analysis studies^15,16^. However, new models are able to track the differential processing of some organic compounds (e.g. amino acids) through the microbial vs. metazoan food webs. Trophic amino acids (as alanine or glutamic acid) are enriched in heavy isotopes with each trophic step, while source amino acids (e.g. phenylalanine), remain essentially unaltered through the food web. Therefore, the comparison of their isotopic composition allows for the estimation of the contribution of heterotrophic protists to the trophic position of metazoan consumers^17,18^. In this way, the pathways of microbial reworking of the dissolved organic matter can be included because protists represent the main link between microbial and metazoan food webs^1–3^. Considering microbial steps will thus produce higher estimations of trophic position, but how this increase in the estimations would affect different consumers along the food web is not known.

Micronektonic fishes are key components of oceanic food webs. They dominate the biomass of consumers^19^ and the transfer of energy from zooplankton to higher trophic levels^20,21^. While their abundance is higher in the mesopelagic domain (200–1000 m depth), the distribution of the different species, including those performing diel vertical migrations also covers the epipelagic (0–200 m), bathypelagic (1000–4000 m) and even abyssopelagic (> 4000 m) layers^22–24^. Therefore, they provide a privileged opportunity to study trophic connections across the ocean. Studies on these organisms focused on changes in their diet and trophic position in relation to their migration habits and vertical distribution. Their trophic position varies within relatively narrow limits as some species are mostly planktivores^20,25–28^ or piscivores^20,25,29,30^, while increasing significantly with individual size^20,25,27^. In addition, there is evidence of different feeding strategies for active migrants vs. opportunistic, non-migrant species^24,30,31^.

As the microbial system is able to recycle several times the produced organic matter, it can be hypothesized that the consideration of microbial steps will have a differential impact on the estimations of trophic position estimates of small and large fishes. The latter are generally predators requiring protein-rich food sources^15^ while the former feed mainly on prey near the base of the food web, hence being more affected by the reprocessing of organic matter by microbial trophic steps^14,18^. The study of the trophic characteristics of micronektonic fishes with different migration habits and vertical distribution may shed some light on the increasing importance of the microbial system with trophic position and individual-size. To examine this hypothesis, we analysed isotope-based trophic indicators of micronektonic fishes in the central region of the N Atlantic to determine the overlap in their trophic niches, their trophic position, and their relative dependence of microbial trophic steps according to their migration habits and vertical distribution.

## Methods

### Sampling

Micronekton fishes were collected in the central N Atlantic during the BATHYPELAGIC cruise (Supplementary Fig. S1). Fish sampling was allowed by the Spanish authorities as part of a research project in international waters. No commercial species were collected and no experiments with living animals were made. Samples were obtained by using a midwater trawl of ca. 30-m^2^ pelagic net equipped with a graded-mesh netting starting with 30 mm and ending with 4 mm^23^ deployed at various depth layers (between the surface and ca. 1900 m) during day and night periods. In this study, samples were combined from different stations and layers to ensure a minimum of three specimens per species for isotopic analysis. Thirteen species of micronektonic fishes were analysed (Supplementary Table S1), including migrants reaching the surface (*Neonesthes capensis*, *Benthosema glaciale*, *Lobianchia dofleini* and *Chauliodus danae*), partial migrants reaching the subsurface layer up to 100 m (*Argyropelecus hemigymnus* and *Photostomias guernei*), and non-migrants always distributed below 250 m (*Bathylagus euryops*, *Cyclothone alba*, *C. braueri*, *C. microdon*, *Sigmops bathyphilus*, *Scopelogadus beanii*, and *Taaningichthys bathyphilus*). These species provided a range of individual sizes between 5 mg and 14 g of dry weight (17 to 275 mm in length). Information on migration habits, depth distribution, depth, and previous estimates of TP was obtained from the literature (see references in Table S1), our own data, and FishBase^32^. For subsequent analysis, the species were grouped according to three layers of vertical habitat: 0–1000 m, 1000–2000 m and > 2000 m (Supplementary Table S1).

Specimens were sorted, identified on board, and kept frozen (− 20 °C). In the laboratory, each fish was first measured as standard length (SL, mm), eviscerated, freeze dried, and weighed (dry weight, DW, mg).

### Stable isotope analysis

Portions of dorsal muscle (where possible) or whole individuals without gut and gonads (for small-sized specimens) were ground and homogenised, and subsequently analysed for δ^15^N and δ^13^C in bulk tissues. Separate determinations were made for each element, using untreated aliquots for δ^15^N, and lipid-extracted portions for δ^13^C. The latter were obtained by the removal of polar and neutral lipid fractions with a mixture of trichloromethane, methanol, and water^33^. Determinations were made using an elemental analyser coupled to an isotope-ratio mass spectrometer and δ^15^N and δ^13^C were computed using international standards^34^. For each analytical run, isotope standards from the International Atomic Energy Agency (IAEA-600, urea, and l-alanine), and internal standards (acetanilide and cyanobacteria of known isotope composition) were employed. Samples were analysed in triplicate. Precision (± s.e.) of replicate determinations of standards and samples for both isotopes was < 0.1 and < 0.4‰, respectively.

Freeze-dried, 10 mg aliquots were also used for determinations of δ^15^N in individual amino acids^35^. Preparation of samples included hydrolysis with 6 N HCl (20 h, 110 °C) and the formation of trifluoroacetyl/isopropyl ester derivatives^36,37^. An internal amino acid standard (l-norleucine) of known isotopic composition was added to each sample. Hydrolysed extracts were filtered by 0.20 μm hydrophilic filters and evaporated to dryness under N_2_ at 60 °C. Esterification was made with 2.5 ml of 1:5 acetyl chloride:2-propanol, flushed with N_2_ and heated to 110 °C for 60 min. For acylation, samples were first dried at ambient temperature under a stream of N_2_, then heated to 110 °C for 15 min with 0.9 ml of 3:1 diclomethane:trifluoracetic anhydride (DCM:TFAA). Derivatized amino acids were purified by solvent extraction in 3 ml of 1:2 chloroform:phosphate buffer (Na_2_HPO_4_ + NaH_2_PO_4_ in Milli-Q water, pH 7.4). The chloroform and the acyl-derivatives were collected in a clean vial after centrifugation (10 min, 17,000×*g*) and evaporated at room temperature under N_2_. Samples were stored at − 20 °C in 3:1 DCM:TFAA for up to 6 months until isotope analysis.

Derivatized products were injected into a mass spectrometer coupled to a gas chromatograph by a continuous flow interface and a combustion module. The individual amino acids were separated using a TraceGOLD TG-5MS chromatographic column (60 m, 0.32 mm ID, 1.0 μm film). The δ^15^N of each amino acid in the sample was calibrated with the values obtained for isolated standards analysed by combustion as described for bulk analysis. Precision (± s.e.) of triplicate samples (two injections per sample) was < 1.5‰ per individual amino acid. The molar fraction of each amino acid was also determined along with δ^15^N by calibration of the spectrometric signals with mixtures of amino acids using both certified and cyanobacterial standards, as for the bulk analysis^36^. Values of δ^15^N were obtained for alanine (Ala), glycine (Gly), threonine (Thr), serine (Ser), valine (Val), leucine (Leu), isoleucine (Ile), proline (Pro), methionine (Met), phenylalanine (Phe), lysine (Lys), and the mixtures of glutamine (Gln) and glutamic acid (Glu), and of aspartamine (Asn) and aspartic acid (Asp). The latter mixtures were caused by the acid hydrolysis and were termed as Glx and Asx, respectively. Following previous studies^35,38,39^ these amino acids were categorized as trophic (Glx, Asx, Ala, Ile, Leu, Pro and Val) and source (Gly, Ser, Lys, Phe, Thr and Met). The representativity of these amino acids for the bulk protein was determined by correlation of their weighted average δ^15^N (δ^15^N_THAA_) with δ^15^N of bulk samples, by taking into account the molar fraction of each individual amino acid^36^. Further comparisons were made also using mean δ^15^N values of trophic and source amino acids using both arithmetic mean and weighted mean values, but only the former were finally reported.

The size and overlap of the isotopic niche defined by bulk δ^15^N and δ^13^C of individual samples was computed with Bayesian statistics using the package NicheROVER^40^. Because of the low number of samples in some of the combinations of migratory habits and layers, separate analyses for migrant vs. non-migrant and for depth layers were made using 10^5^ simulations. This analysis allowed the estimation of asymmetric probabilities of overlap between niche categories.

Trophic positions (TP) were estimated from CSIA results by taking into account the variability of trophic discrimination factors (TDF) accounting for the enrichment in δ^15^N with each trophic step. The general multi-TDF model of McMahon and McCarthy^39^ was applied to different trophic amino acids (Glx or Ala) and Phe as the source amino acid:

1 $${\text{TP}}_{{{\text{Tr}}}} = { 2 } + \, \left( {\delta^{{{15}}} {\text{N}}_{{{\text{Tr}}}} - \, \delta^{{{15}}} {\text{N}}_{{{\text{Phe}}}} {-}{\text{ TDFp}}_{{{\text{Tr}}}} \beta_{{{\text{Tr}}}} } \right)/{\text{TDFf}}_{{{\text{Tr}}}} ,$$

where subindex Tr refers to Glx or Ala, β is the difference between Tr (Glx or Ala) and Phe in primary producers, and TDFp_Tr_ and TDFf_Tr_ are the trophic discrimination factors for plankton and fish, respectively. TP_Glx_ is an estimate of the trophic position by taking into account only metazoan steps, while TP_Ala_ considers microbial + metazoan steps^17,18^. Different β and TDF values (mean ± s.d.) were applied for plankton^18,35^, and for teleost fish^41^ (β_Glx_ = 3.6 ± 0.5‰; β_Ala_ = 3.2 ± 1.2‰; TDFp_Glx_ = 7.6 ± 1.2‰; TDFf_Glx_ = 5.7 ± 0.3‰; TDFp_Ala_ = 4.5 ± 2.1‰; TDFf_Ala_ = 6.1 ± 0.3‰).

The propagated error (s.d.) in the estimation of TP was calculated by taking into account the analytical errors in the individual trophic (Glx or Ala) and source amino acids (Phe), as well as the variability in the β and TDF values employed^41^. The fractional contribution of microbial steps to TP_Ala_ was computed as:

2 $$\% {\text{ Microbial }} = { 1}00 \, \left( {{\text{TP}}_{{{\text{Ala}}}} {-}{\text{ TP}}_{{{\text{Glx}}}} } \right)/{\text{TP}}_{{{\text{Ala}}}} .$$

Alternatively, TP could have been estimated using a single TDF^29^ or mean values for trophic and source amino acids^16,41^ but the multi-TDF model improved the correspondence between isotope-derived and gut-content derived TP^39^. Moreover, the estimation of the microbial contribution would not have been possible when averaging trophic amino acids.

Differences between species grouped by migration habits and depth layers were assessed using PERMANOVA and non-parametric ANOVA (Kruskal–Wallis). Paired post-hoc tests (Bonferroni, Tukey) were applied to identify significant differences between groups. Regressions between variables were computed using product-moment regression (when there were large differences in their errors) or reduced major axis regression (when the errors in the variables were of similar magnitude). Statistical analyses were made using SPSS 17.0 (SPSS Inc.) and Past 4.0^42^.

## Results

### Niche overlap

**Figure 1:**
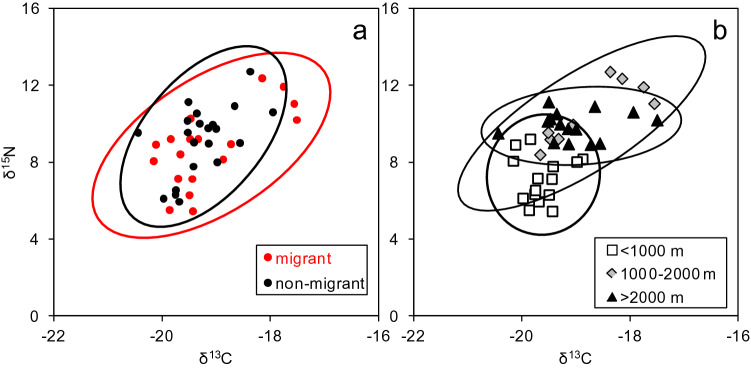
Plot of bulk δ^15^N and δ^13^C grouped by migratory (**a**) or depth layer distribution characteristics (**b**). The ellipses encompassing 95% of data for each group are indicated.

Values of stable isotopes in bulk samples varied significantly between depth distribution layers, but not according to the migration habits (see Supplementary Tables S1, S2). Mean bulk δ^15^N and δ^13^C values for species distributed in the intermediate and deep layers were higher than those in the upper layer, with similar values for migrant and non-migrant species (Supplementary Fig. S2). Consequently, there was a large coincidence in the isotopic niche of migrants (and partial migrants) and non-migrants (Fig. 1a), and a much clearer separation of niches by depth layers (Fig. 1b). The estimated mean probability of overlap between the non-migrants niche with that of migrants exceeded 90%, and the probability of a migrant species to be found in the niche of non-migrants was ca. 80% (Supplementary Fig. S3a). In contrast, the niche of the species in the upper layer had mean overlap probabilities of < 40% and < 20% with the intermediate and deep layers, respectively (Supplementary Fig. S3b). Even lower values were obtained for the converse overlap. However, large overlap (> 60%) was observed between the niche of the species living in the intermediate and deep layers.

### Trophic and source amino acids

The values of δ^15^N of total hydrolysable amino acids, as well as mean values for trophic and source amino acids, were significantly correlated with δ^15^N in bulk samples, and these relationships were not affected by migration habits or layer distribution (Supplementary Fig. S4). The higher correlation values for δ^15^N in trophic vs. source amino acids indicate that bulk δ^15^N mainly reflected trophic positions.

**Figure 2:**
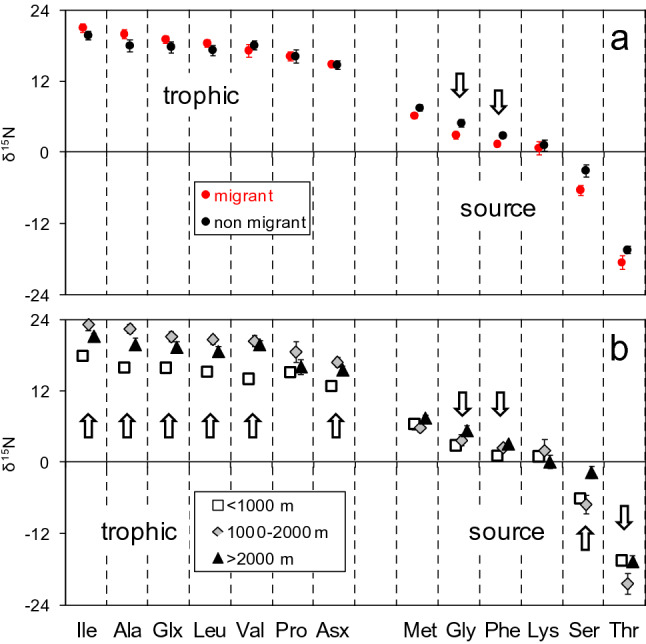
Mean (± s.e.) δ^15^N (‰) of trophic and source amino acids for (**a**) migrant (black dots) and non-migrant (red dots) species, and (**b**) for species grouped by depth layers. The arrows indicate significantly different means (Kruskal–Wallis test, P < 0.05).

As for bulk δ^15^N, there were no significant differences in mean values of δ^15^N in trophic amino acids when species were grouped by migration habits (Supplementary Table S2), but non-migrant species had significantly higher values in source amino acids Gly and Phe (Fig. 2a, Supplementary Table S2). In contrast, δ^15^N values for almost all trophic amino acids were higher in species from the intermediate and the deeper layers than in those from the upper layer (Fig. 2b). Source amino acids Gly and Phe also showed high δ^15^N values in the deeper layers, while those for Ser and Thr were only significant for the deep or intermediate layer, respectively. In spite of these differences in source amino acids, there was no significant variation across the latitude gradient of samples, as exemplified by δ^15^N_Phe_ (Supplementary Fig. S5).

### Trophic positions

**Figure 3:**
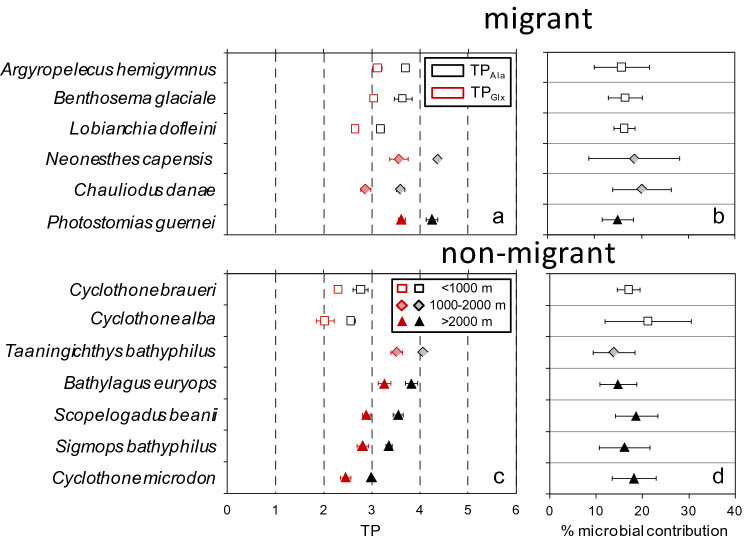
Mean (± s.d.) trophic positions (**a**,**c**) and microbial contribution (**b**,**d**) including microbial + metazoan (TP_Ala_) or only metazoan (TP_Glx_) food webs for migrant (**a**,**b**) or non-migrant (**c**,**d**) species coded by habitat depth. The values of integer TP are indicated by dashed lines.

The studied species showed mean trophic positions between 2.56 and 4.36, when considering the full food web (TP_Ala_, Fig. 3a,c). The lowest values were for the non-migrant, mesopelagic- dwelling, zooplanktivore *Cyclothone alba*, and the highest for the migrant, micronektivore *Neonesthes capensis*. Taking into account the microbial food web (TP_Ala_) increased the mean estimations between 0.5 and 0.8 TP. These differences were higher than the errors of the estimations (mean values of 0.1 TP for both TP_Ala_ and TP_Glx_) and imply contributions of microbial steps to the total TP between 14 and 21% (Fig. 3b,d). Migrant species and species distributed below 1000 m had significantly higher TP values than those of non-migrants and living in the upper layer, but the differences between TP_Ala_ and TP_Glx_ did not vary significantly with migration or between layers (Supplementary Table S3).

**Figure 4:**
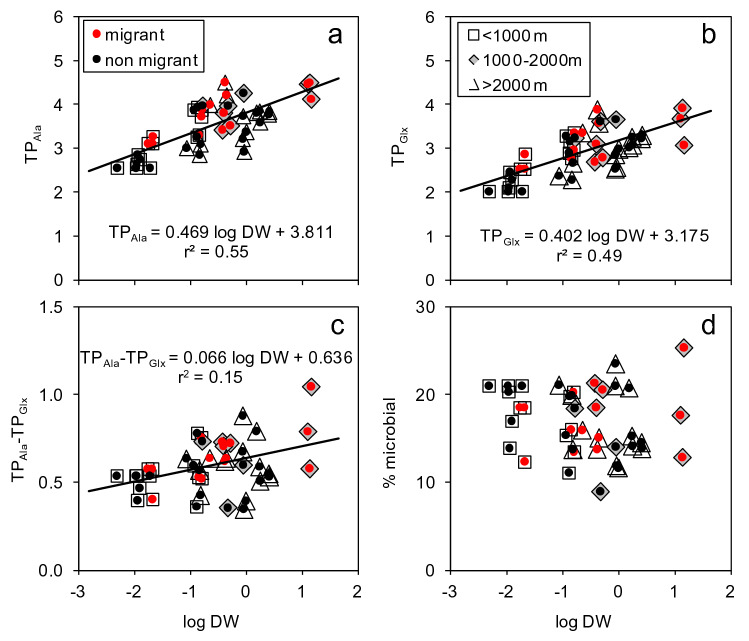
Relationships between trophic positions including (**a**) microbial + metazoan (TP_Ala_) or (**b**) only metazoan (TP_Glx_) food webs and (**c**) their difference (TP_Ala_ − TP_Glx_) or (**d**) the fractional contribution of microbial food web (%microbial) with log-transformed individual dry weight (DW, mg). As the slopes for migrant (black dots) and non-migrant (red dots) were no significantly different (ANCOVA, P > 0.05), the regression lines for the whole data are indicated (P < 0.001) along with the corresponding determination coefficients (r^2^).

Both TP estimations increased significantly with the log of individual weight (Fig. 4a,b), but the slope of the regression was higher for TP_Ala_ than for TP_Glx_, thus implying that the difference between both estimations also increased with body weight (Fig. 4c). However, when scaled to the TP_Ala_ values, the microbial contribution was neither related to body weight (Fig. 4d) or to other factors, such as species, taxonomic order, or diet (Supplementary Table S4). No significant effects of migration habits or depth layers were found to affect these relationships, either.

## Discussion

This study shows that niche separation in micronektonic fishes is due to the vertical habitat range rather than to migration. The large niche overlap of migrant and non-migrant species can be expected because the feeding strategies of the species take advantage of different resources in each depth layer^24,30,31^. In turn, differences in stable isotopes between depth layers can be attributed to the vertical separation of resources, as δ^15^N and δ^13^C increase with depth consistently with a major reworking of the settling organic matter^43^. Previous studies also reported a vertical change in the source amino acids, and particularly in δ^15^N_Phe_, for zooplankton^44^ and micronektonic fishes^45^, thus supporting the exploitation of resources in deep layers^26,28,30,45^. In this context, vertical migration alleviates trophic competition by allowing the access to different food resources while the niche overlap was most likely between adjacent layers. The largest niche separation was found between the species distributed above and below 1000 m depth. The estimated trophic positions were also higher for species distributed in the deep layers irrespective of their migratory habits. These results suggest the existence of a major vertical limit for the influence of epipelagic vs. bathypelagic food webs. It can be argued that the high energetic costs and predation risks of migration from deep layers^46^ must be compensated by the access to the abundant prey in the epipelagic layer. However, this requirement may be not always met in central areas of the ocean where the abundance of zooplankton is lower than near the coast^9,47^. Indeed, there are indications of the existence of an active food web in bathypelagic waters supported by settling organic matter^29,45,47,48^ but also by in situ production by microorganisms^49–51^. The latter implies an increase of the links between metazoan and microbial food webs that is partly supported by the observed isotopic enrichment in particles and organisms^26,44,45^.

Our estimates of species-specific trophic position using amino acid δ^15^N, but particularly TP_Ala_, agree with those available in the literature and based on gut contents (see Supplementary Fig. S6). Previous studies suggested the use of multi-trophic isotopic models to improve the correspondence between both types of estimates to take into account the quality of the consumed food and the excretion products^52,53^. In our study, we showed that the consideration of the microbial trophic steps produced TP values almost equivalent to those derived from stomach contents, while TP based only on the metazoan food web were systematically lower. In addition, three species (*N. capensis*, *Chauliodus danae*, and *Photostomias guernei*) considered as micronektivores because of the presence of small fish and crustaceans in their stomachs did not always have the highest TP when estimated from amino acid δ^15^N. These results suggest that the diet of these species is far more complex and variable than indicated by gut content analysis, missing most of the components of the microbial food web. This calls for a review of the current assignation of trophic position values to prey items based on an established convention of TP = 1 for primary producers and detritus and TP = 2 for zooplankton^54,55^. Indeed, it is possible that part of the apparent correspondence between TP estimates in FishBase and TP_Ala_ values would be a spurious result caused by the uncertainties in the assignation of TP to the identified prey in the former.

The calculated TP values are also within the range of estimates obtained using stable isotopes, even taking into account the uncertainties associated with the selection of appropriate baselines and trophic enrichment factors in case of bulk determinations. For instance, TP values for *Benthosema glaciale*, *Lobianchia dofleini*, *C. alba* and *Argyropelecus hemigymnus* were within the range estimated from bulk δ^15^N for these species in the Atlantic^27,56^, and those for *B. glaciale*, *A. hemigymnus* and *Cyclothone braueri* agreed with TP estimates in the Mediterranean^25^. The main difference was for *L. dofleini*, that in the present study was ca. 1 TP lower, probably due to the smaller sizes of specimens analysed (32.0 mm in the Mediterranean vs. 17.7 mm in this study). Larger coincidence was found for TP estimations using also amino acid δ^15^N values, as in the case of *A. hemigymnus* in the Gulf of Mexico^28^. These results suggest that, despite regional differences in resource availability, most species show consistent trophic positions varying within relatively narrow limits. This is supported by the growing number of TP estimations based on δ^15^N in amino acids, accounting for local and regional differences in the isotopic signature of the sources of nitrogen for primary producers^26,28–30,45^.

In our study, migrant fishes had higher trophic position values than non-migrants, but both migrant and non-migrant species distributed in the bathypelagic layer had also higher TP than those in the upper layer. Other studies also noted this increase in TP with migration and depth distribution which was attributed to the access to a larger variety of prey and the predominance of piscivory in deep layers^24,25,28,30^. Correspondingly, our metazoan TP estimates for species distributed in the bathypelagic layer were always above 2.5, indicating they were secondary consumers, although the values for the gonostomatid *Cyclothone microdon* (mean ± s.d. = 2.45 ± 0.11) suggested a mixed diet including zooplankton, as observed in direct dietary analyses (see Supplementary Table S1). Among the stomiiforms studied we observed lower TP in the mesopelagic species, *C. alba* and *C. braueri*, than in the bathypelagic *C. microdon* and *Sigmops bathyphilus*. Our results also show that the reported higher TP in species of the family Stomiidae compared to those in Myctophidae^29^ may not be a universal feature, and may depend on the species, sizes and depth range. For instance, we found TP > 3.5 for the three Stomiidae examined (*N. capensis, C. danae* and *P. guernei*), while the bathypelagic myctophid *Taaningichthys bathyphilus* (for which there is no published data on stomach content) had a mean ± s.d. TP of 4.07 ± 0.09. The comparison between our TP estimates with those derived from diet analysis also revealed that those species ranked as piscivores (see Supplementary Table S1) were not always those with the highest TP (e.g. *Chauliodus danae*), thus reflecting a large plasticity in the diets of these fishes. As the composition of the micronekton community is more complex than suggested by the simplification to a few groups^23,57^, we also include representatives of orders Stephanoberyciformes (*Scopelogadus beanii*) and Argentiniformes (*Bathylagus euryops*) among the selected species. These species are consumers of gelatinous plankton (see Supplementary Table S1) and ther estimated TP, ca. 3, is similar to those for other species consuming prey richer in protein than gelatinous plankton. Thus, these relatively high values may in fact reflect the deep habitat of the species, which may be the case of *S. beanii*, reported to feed on salps^58^ or they could also be explained by the consumption of predatory prey, since species of *Bathylagus* showed a substantial fraction of siphonophore DNA in their stomachs^59^.

Nevertheless, the estimations of trophic position based only on metazoan steps were lower than those considering also microbial steps with an average microbial contribution to overall TP of 17%. Some species (as those of genus *Cyclothone*) had relatively large variation in the microbial contributions that could be attributed to their feeding on detrital aggregates (see Supplementary Table S1). These results are quite similar to the only published estimations of microbial contributions to pelagic food webs, which use the same methodology, but applied to epipelagic zooplankton^18^. These, indicate microbial contributions up to 35% in omnivorous species and between 0 and 11% for herbivores. Fatty acid markers also revealed a significant dietary contribution of bacterial organic matter to some subtropical copepods^48^. The prevalence of microbial contributions in planktivores and piscivores found in this study highlights the importance of detritus degradation and microbial reworking particularly in deep layers of the ocean. In the absence of phytoplankton, a better use of the organic matter in the bathypelagic domain can be made through trophic steps involving chemoautolithotrophy^49,50^, uptake of dissolved organic matter by bacteria^51^, and protozoan grazing on bacteria^8,60^, among those within the microbial food web. Further transfer of the organic matter concentrated by the microbial food web to the metazoan consumers is made by zooplankton^61,62^ and, as shown in our study, by micronekton fishes. Even when the main contribution to the top consumers of the food web (e.g. sharks, tunas, birds, cetaceans) requires a substantial contribution of direct transfers of primary production through the metazoan food web^2,54^, the estimations made in our study suggest that, at least in the oligotrophic regions of the ocean, the contribution of the microbial food web cannot be neglected.

The increase in amino acid based TP with the logarithm of individual size confirms that pelagic food webs are size-structured^63^, as already found in studies using bulk δ^15^N^12,64–66^. This structure implies that the trophic efficiency decreases with size as the ratio between the sizes of predator and prey increases^67^ thus placing an upper limit to the number of trophic levels that can be sustained in a food web^68,69^. Meta-analysis of aquatic food web studies concluded that marine pelagic ecosystems, even having a higher number of trophic levels than freshwater ecosystems, will not have more than 5 trophic levels^68,70^. The required energy conversions and losses in models based on biomass propagation may lead to the underrepresentation of multiple or alternative trophic pathways. However, other analyses revealed that top predators TP were underestimated, notably by conventional models based on bulk δ^15^N^15^. In addition, estimations using the biomass spectrum suggested that the total number of trophic levels could increase because of the omnivorous and opportunistic feeding of zooplankton^71^. Zooplankton, indeed, represents the link between the microbial and metazoan food webs supporting higher trophic levels and vertical export^62^. The addition of more trophic levels by taking into account the microbial steps supports the increase of TP estimates, at least for zooplankton^18^ and micronekton (this study).

Taking into account microbial steps, TP of the micronekton fishes studied increased linearly with the logarithm of body weight. However, we did not find significant effects for size, taxonomical categories, migration habits or vertical distribution on the relative importance of the microbial food web when scaled to TP values. Our estimates varied between relatively narrow limits (9–25%) comparable to those reported for zooplankton^18^ and can be attributed to the widespread omnivory of these organisms. In the case of crustacean zooplankton there was a clear increase in the microbial contribution in omnivores, but the absence of a clear pattern in planktivorous and piscivorous fishes suggests the existence of an upper limit to the contribution of the microbial steps to the trophic position. In fact, food webs may exhibit a high number of trophic links with low energetical importance while only a small number of links influence the transfer of energy and biomass to upper trophic levels^69,72^. Besides, the growth efficiency of pelagic bacteria is generally low in the deep ocean because of large respiratory losses^4,73^. In this way, we could expect that the contribution of metazoan components to the overall TP should increase for the upper trophic levels. However, further studies including other organisms from the food web will be required to confirm this hypothesis. Our study showed that TP estimations of mid-trophic level consumers can be improved by taking into account the contribution of microbial trophic links, suggesting that current estimations of TP and food chain length need to be revised.

## Supplementary Information

Supplementary Information.

## Data Availability

The original data on sample location, individual fish characteristics and stable isotope composition, including amino acids, can be accessed through the PANGAEA repository (10.1594/PANGAEA.930111).
