# Supplementary material for: Trophic indices for micronektonic fishes reveal their dependence on the microbial system in the North Atlantic
Source: Sci Rep. 2021 Apr 19;11:8488. doi: 10.1038/s41598-021-87767-x (PMC8055700; doi:10.1038/s41598-021-87767-x)
Supplement: Supplementary file 1 — Supplementary Information. [file 41598_2021_87767_MOESM1_ESM.pdf]

Trophic indices for micronektonic fishes reveal their dependence on the microbial system in the North Atlantic

Antonio Bode, M. Pilar Olivar, Santiago Hernández-León

Supplementary information

Supplementary Table S1

Supplementary Table S2

Supplementary Table S3

Supplementary Table S4

Supplementary Figure S1

Supplementary Figure S2

Supplementary Figure S3

Supplementary Figure S4

Supplementary Figure S5

References

Supplementary Table S1. Mean (± s.e.) values of $\delta^{13}C$ (lipid-free) and $\delta^{15}N$ (‰) of bulk ($\delta^{13}C_{bulk}$ and $\delta^{15}N_{bulk}$), source ($\delta^{15}N_{src}$), and trophic amino acids ($\delta^{15}N_{trp}$) for three samples for each species studied. The standard length (SL, mm) and eviscerated dry weight (DW, mg) values of the analised specimens, along with their potential depth range (m) and daily migration (M: migrant, PM: partial migrant, NM: non migrant) are also indicated. NA: information not available.

species	depth range	migrant	diet type	SL	DW	$\delta^{13}C_{bulk}$	$\delta^{15}N_{bulk}$	$\delta^{15}N_{src}$	$\delta^{15}N_{trp}$
Neonesthes capensis	70-1650[1]	M	micronektonivore[1]	254.3 ± 10.5	13397.3 ± 525.0	-17.82 ± 0.17	11.76 ± 0.39	0.56 ± 0.49	22.66 ± 0.89
Bathylagus euryops	500-3000[2]	NM	zooplanktivore (gelatinous prey)[3,4]	120.3 ± 3.2	2250.0 ± 274.1	-19.77 ± 0.34	10.38 ± 0.46	3.35 ± 0.41	19.95 ± 0.23
Cyclothone alba	300-600[5]	NM	zooplanktivore - detritivore[4]	23.0 ± 2.1	11.3 ± 4.0	-19.54 ± 0.29	6.67 ± 0.66	0.19 ± 0.01	13.87 ± 1.00
Cyclothone braueri	250-900[5]	NM	zooplanktivore - detritivore [6]	24.3 ± 0.3	11.4 ± 0.4	-19.63 ± 0.11	6.86 ± 0.45	1.20 ± 0.94	13.49 ± 0.56
Cyclothone microdon	500-2700[5]	NM	zooplanktivore- detritivore[7]	44.0 ± 0.6	126.0 ± 21.1	-19.27 ± 0.13	9.61 ± 0.34	2.47 ± 0.37	18.28 ± 0.57
Sigmops bathyphilus	700-3000[5]	NM	NA	131.0 ± 3.5	909.5 ± 35.0	-18.54 ± 0.31	10.41 ± 0.35	1.26 ± 3.49	16.60 ± 4.20
Scopelogadus beanii	800-2500[8]	NM	zooplanktivore (gelatinous prey)[4,9]	79.3 ± 3.2	1365.2 ± 265.2	-19.09 ± 0.27	9.33 ± 0.33	3.14 ± 0.75	17.17 ± 0.26
Benthosema glaciale	0-850[10]	M	zooplanktivore[6]	38.3 ± 1.5	150.9 ± 5.4	-19.59 ± 0.14	5.74 ± 0.27	-0.50 ± 0.17	15.13 ± 0.47
Lobianchia dofleini	0-700[10]	M	zooplanktivore[6]	17.7 ± 0.3	19.6 ± 1.2	-20.03 ± 0.10	8.70 ± 0.34	0.14 ± 0.26	15.94 ± 0.01
Taaningichthys bathyphilus	675-1750[10]	NM	NA	60.3 ± 8.2	500.3 ± 206.3	-18.98 ± 0.34	10.72 ± 0.99	2.30 ± 1.50	21.28 ± 1.25
Argyropelecus hemigymnus	100-800[11]	PM	zooplanktivore[6]	32.7 ± 0.7	124.2 ± 5.7	-19.34 ± 0.24	7.46 ± 0.34	1.33 ± 0.94	17.94 ± 1.06
Chauliodus danae	0-1600[12]	M	micronektonivore (fish)[13,14]	101.3 ± 0.7	422.4 ± 43.8	-19.49 ± 0.09	8.93 ± 0.26	2.37 ± 1.33	17.65 ± 0.49
Photostomias guernei	100-3000[15]	PM	micronektonivore (crustaceans)[14]	102.7 ± 6.4	353.8 ± 67.3	-18.57 ± 0.57	9.79 ± 0.43	2.07 ± 1.32	19.76 ± 0.43

Superscript numbers indicate references listed at the end of this supplement

Supplementary Table S2. Significance of factors: migration (migrants + partial migrants, non migrants) and depth layer (0-1000 m, 1000-2000 m and > 2000 m) tested using two-way PERMANOVA for different isotope variables. The number of data points for each combination of factors varied between 3 and 12. Significant values ($P<0.05$) are indicated in bold. $\delta^{13}C_{bulk}$ and $\delta^{15}N_{bulk}$: $\delta^{13}C$ (lipid-free) and $\delta^{15}N$ (‰) of bulk samples, respectively; $\delta^{15}N_{trp}$ and $\delta^{15}N_{tsrc}$: mean $\delta^{15}N$ (‰) of trophic and source amino acids, respectively; TP_{Ala} and TP_{Glx}: mean trophic positions including microbial +metazoan or only metazoan food webs, respectively; TP_{Ala}-TP_{Glx}: difference between TP_{Ala} and TP_{Glx}.

variable	migration	layer	migration x layer
$\delta^{15}N_{bulk}$	0.246	**0.000**	0.252
$\delta^{13}C_{bulk}$	0.538	**0.004**	0.187
$\delta^{15}N_{trp}$	0.511	**0.000**	**0.012**
$\delta^{15}N_{src}$	**0.002**	**0.000**	0.715
TP_{Ala}	**0.000**	**0.000**	**0.004**
TP_{Glx}	**0.001**	**0.000**	**0.000**
TP_{Ala}-TP_{Glx}	0.065	0.124	0.705

Supplementary Table S3. Mean (± s.d.) trophic positions including microbial + metazoan (TP_{Ala}) or only metazoan (TP_{Glx}) food webs, and difference between trophic positions by migratory habits and depth layers. n: number of data. Significant values (two-way PERMANOVA, Tukey post hoc test, P<0.05) are indicated with different letters for migratory habits (a, b) or layers (x, y, z).

type	depth layer	TP_{Ala}	TP_{Glx}	TP_{Ala}-TP_{Glx}	n
migrant(*)	0-1000 m	3.50 ± 0.15 x	2.93 ± 0.13 x	0.57 ± 0.11 x	9
	1000-2000 m	3.98 ± 0.16 x	3.21 ± 0.18 x	0.76 ± 0.18 x	6
	>2000 m	4.24 ± 0.07 x	3.61 ± 0.07 x	0.63 ± 0.07 x	3
	all layers	3.78 ± 0.18 a	3.14 ± 0.17 a	0.64 ± 0.16 a	18
non migrant	0-1000 m	2.66 ± 0.13 z	2.15 ± 0.12 z	0.50 ± 0.09 x	6
	1000-2000 m	3.95 ± 0.09 x	3.39 ± 0.13 x	0.56 ± 0.09 x	6
	>2000 m	3.29 ± 0.28 y	2.71 ± 0.26 y	0.58 ± 0.22 x	9
	all layers	3.30 ± 0.32 b	2.75 ± 0.30 b	0.55 ± 0.24 a	21

(*) includes partial migrants

Supplementary Table S4. Results of PERMANOVA tests on the effects of species (13 species), taxonomic order (Stomiiformes, Myctophiformes, Argentiniformes, Stephanoberyciformes), diet (planktivorous, piscivorous), migration habits (migrants + partial migrants, non-migrants) or distribution layer (0-1000 m, 1000-2000 m, >2000 m) on the fractional contribution of microbial steps to species TP (%microbial). SS: sums of squares, F: variance ratio, p: significance.

factor	total SS	within-group SS	F	p
species	565.8	393.3	0.951	0.511
order	565.8	513.3	1.195	0.332
diet	565.8	526.2	2.785	0.107
migration	565.8	565.8	0.000	0.984
layer	565.8	563.8	0.066	0.940

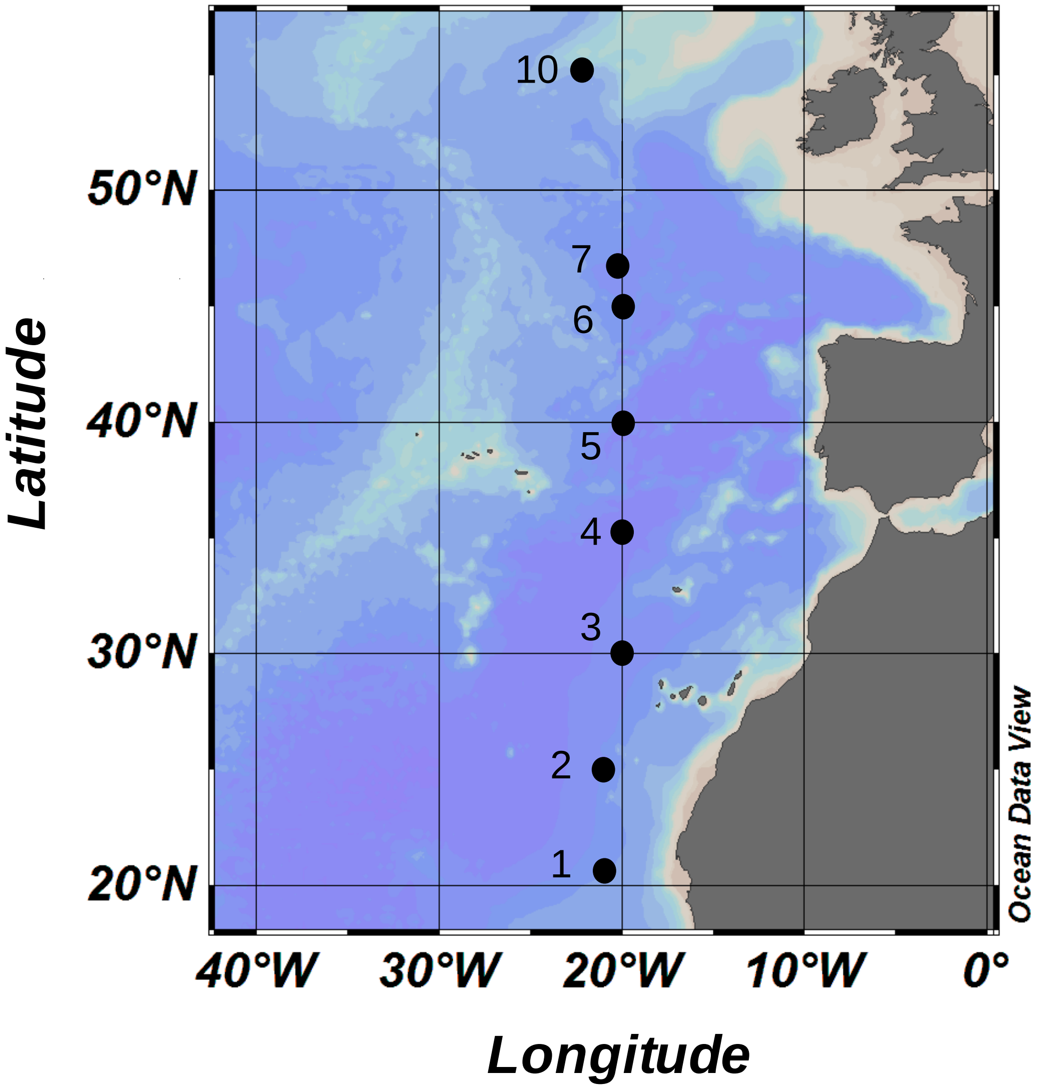

Supplementary Figure S1. Location of micronekton sampling stations for Bathypelagic cruise. Map was created with Ocean Data View v. 4.0.4 (https://odv.awi.de/) [16]

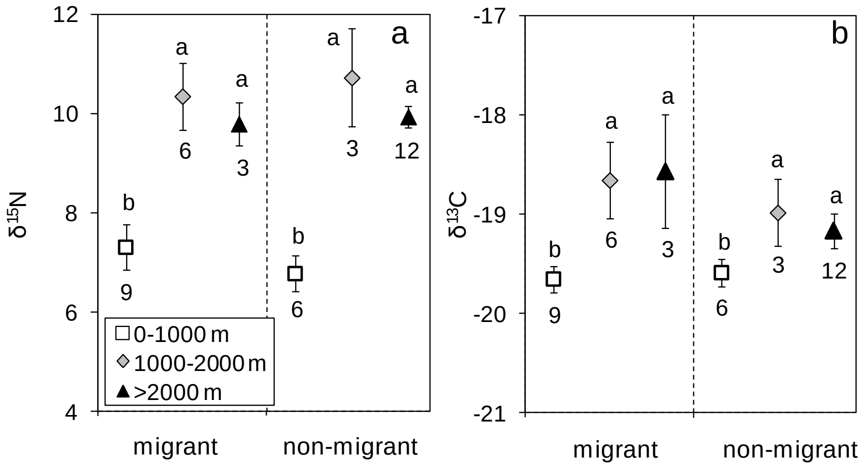

Supplementary Figure S2. Mean (± s.e.) values of bulk (a) $\delta^{15}N$ and (b) $\delta^{13}C$ (‰) for (a) migrant (including partial migrants) and non-migrant species grouped by depth layers. The letters indicate significantly different means (PERMANOVA and post-hoc Bonferroni tests, P<0.05) and the figures the number of data for each category.

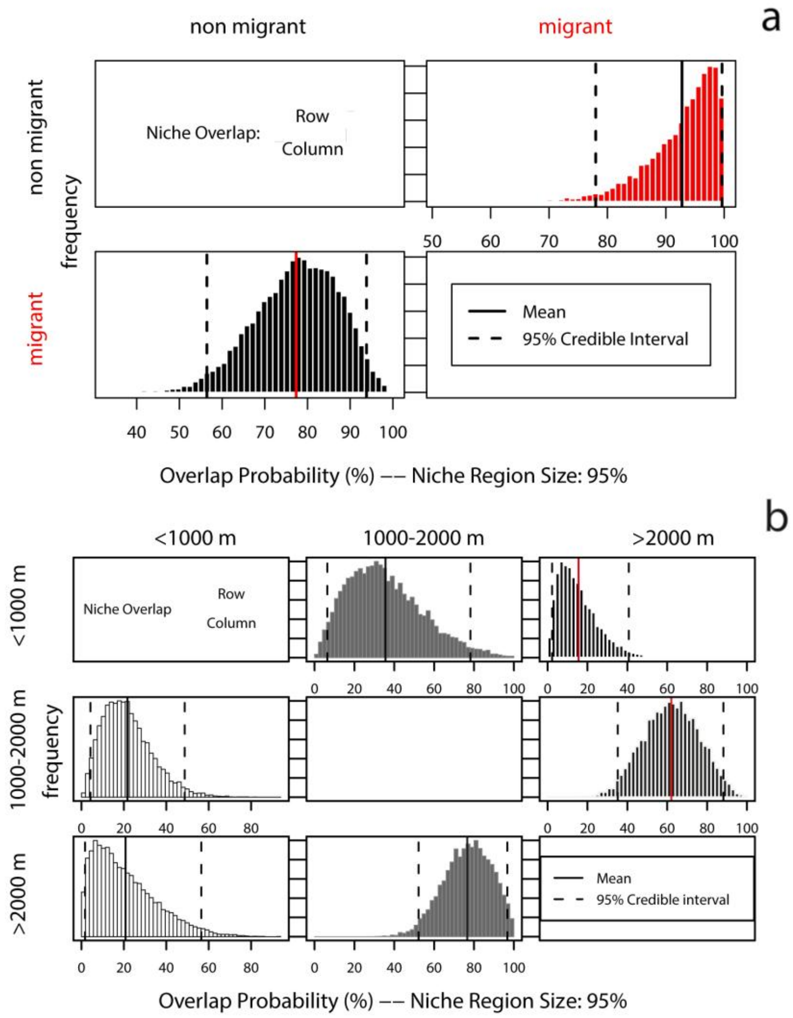

Supplementary Figure S3. Probability of overlap between the Bayesian estimates of isotopic niches of micronektonic fish species grouped by (a) migratory habits, and (b) by three depth layers of potential distribution. The vertical lines indicate the mean (continuous line) and 95% credible intervals (dashed line). Note that the overlap is asymmetric: each plot indicates the probability for an individual of the row category to be included in the column category.

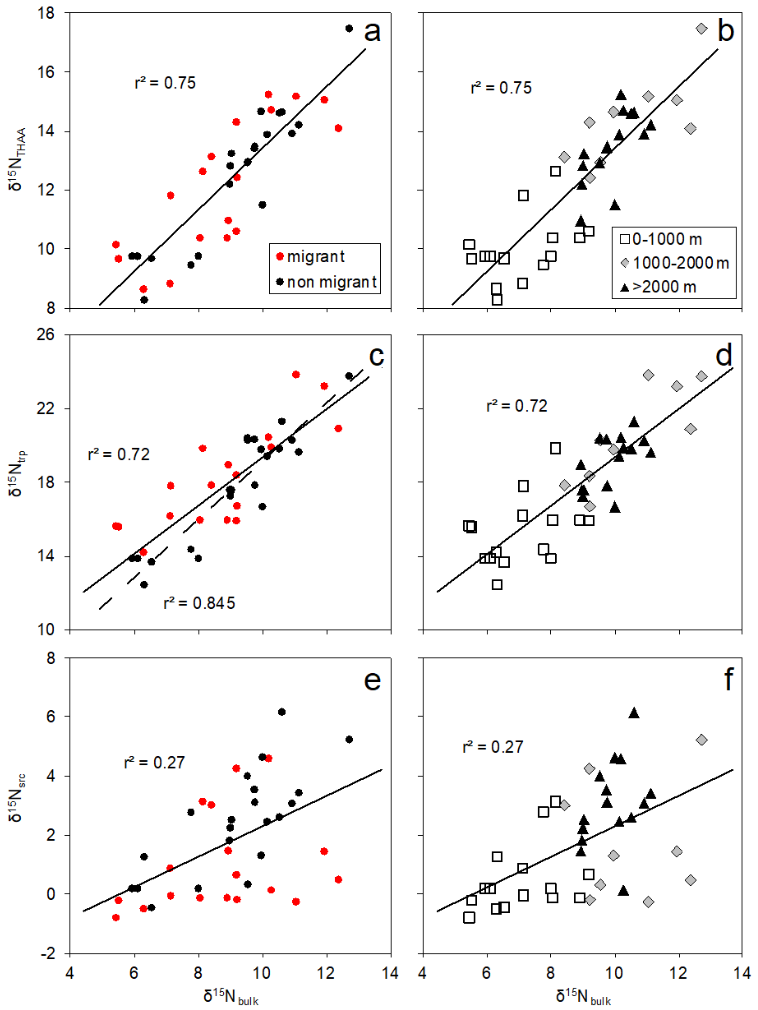

Supplementary Figure S4. Relationships between nitrogen isotope abundance (‰) in bulk samples ($\delta^{15}N_{bulk}$) and in (a, b) total hydrolisable amino acids ($\delta^{15}N_{THAA}$), (c, d) mean value of trophic amino acids ($\delta^{15}N_{trp}$), and (d, e) mean value of source amino acids ($\delta^{15}N_{src}$). The continuous lines indicate the regression lines for all data and the dashed line the regression line only for non migrant fishes ($P<0.001$). r^2: determination coefficient. Values of $\delta^{15}N_{trp}$ and $\delta^{15}N_{src}$ correspond to arithmetic means of individual amino acid values while $\delta^{15}N_{THAA}$ are the mass-weighted means. The use of mass-weighted means produced lower r^2 values but the linear regressions were still significant.

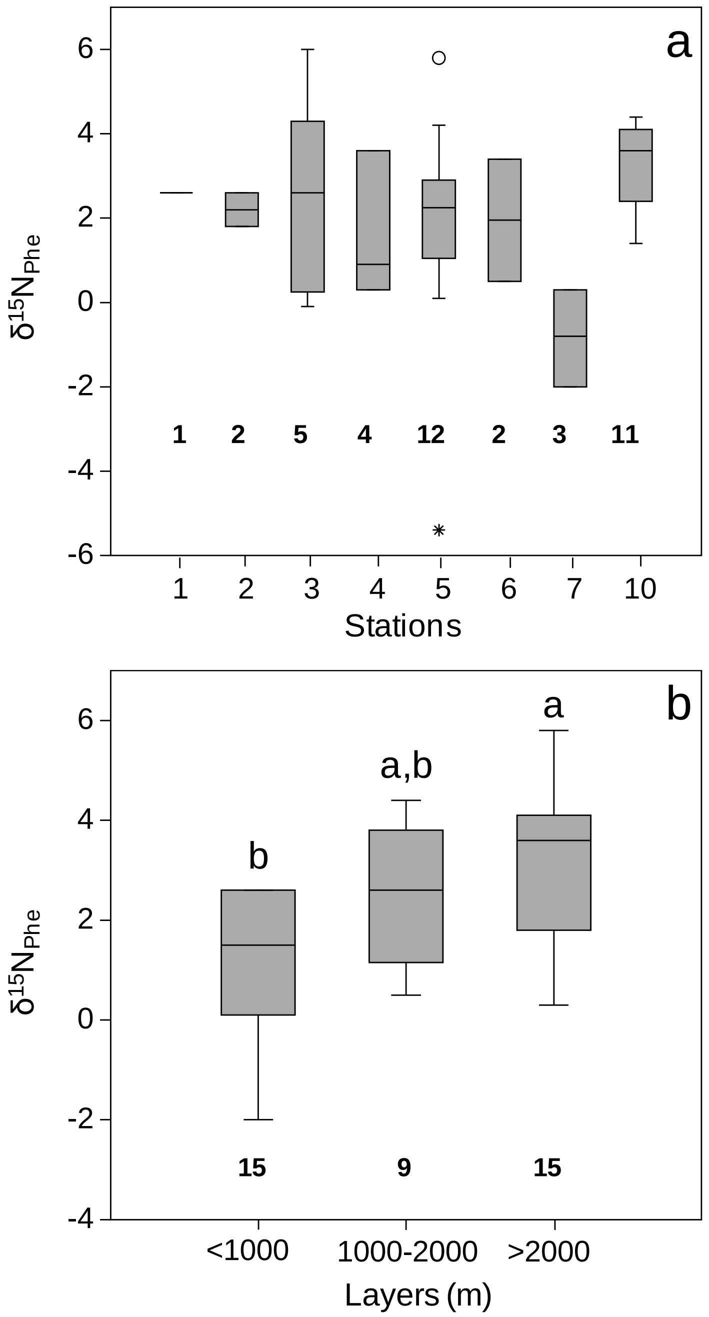

Supplementary Figure S5. Box plot of $\delta^{15}N_{Phe}$ by (a) stations or (b) habitat depth layers. Each box encompassed the 25-75% percentiles, the median is indicated by the horizontal line, and the whiskers indicate the range excluding outliers exceeding 1.5 (circles) or 3 times (asterisks) the interquartile range. The letters above the bars indicate significantly different means (ANOVA and post-hoc Bonferroni test, $P<0.05$) and the number of data is indicated by numbers in bold below each bar. The outliers in panel a were excluded in panel b.

.

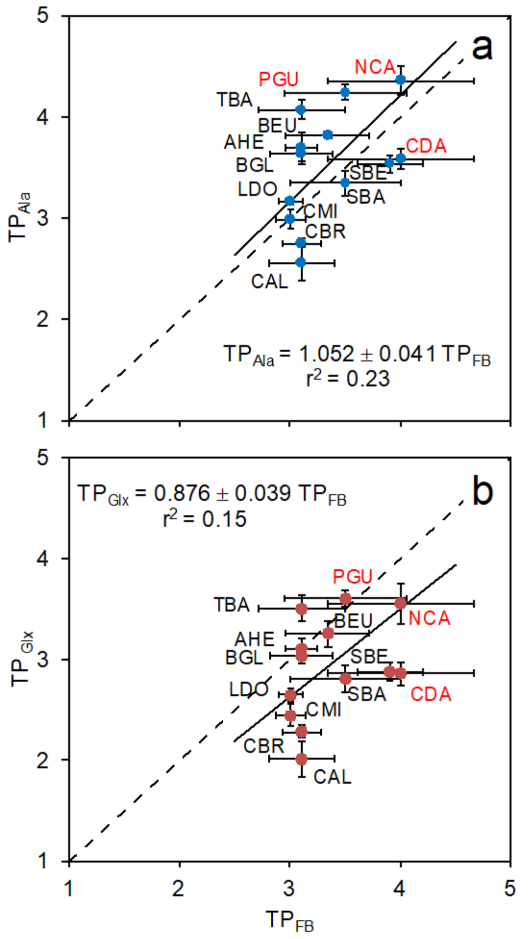

Supplementary Figure S6. Comparison of mean (± s.d.) values of trophic positions from FishBase (TP_{FB}) with estimations considering (a) microbial + metazoan (TP_{Ala}) or (b) only metazoan (TP_{Glx}) food webs. The 1:1 correspondence is indicate by the dashed lines. Reduced major axis regression lines through the origin are indicated by continuous lines (P<0.001). r^2: determination coefficient. Piscivorous species names (according to Supplementary Table S1) are indicated in red. AHE: *Argyropelecus hemigymnus*, BEU: *Bathylagus euryops*, BGL: *Benthosema glaciale*, CAL: *Cyclothone alba*, CBR: *Cyclothone braueri*, CDA: *Chauliodus danae*, CMI: *Cyclothone microdon*, LDO: *Lobianchia dofleini*, NCA: *Neonestes capensis*, PGU: *Photostomias guernei*, SBA: *Sigmops bathyphilus*, SBE: *Scopelogadus beanii*, TBA: *Taaningichthys bathyphilus*.

References

1. Gibbs, R.H.Jr. Astronesthidae in *Fishes of the north-eastern Atlantic and the Mediterranean* (eds. Whitehead, P.J.P., Bauchot, M.-L., Hureau, J.-C., Nielsen, J. & Tortonese, E.) Vol. 1 325-335 (UNESCO, 1984).

2. Sweetman, C.J., Sutton, T.T., Vecchione, M. & Latour R.J. Distribution of the biomass-dominant pelagic fish, *Bathylagus euryops* (Argentiniformes: Microstomatidae), along the northern Mid-Atlantic Ridge. *Deep-Sea Res.* **78**, 16–23 (2013).

3. Sweetman C.J., Sutton, T.T., Vecchione, M. & Latour R.J. Diet composition of *Bathylagus euryops* (Osmeriformes: Bathylagidae) along the northern Mid-Atlantic Ridge. *Deep-Sea Res.* **92**, 107–114 (2014).

4. Drazen, J.C. & Sutton, T.T. Dining in the Deep: The Feeding Ecology of Deep-Sea Fishes. *Annu. Rev. Mar. Sci.* **9**, 337-366 (2017).

5 Badcock, J. Gonostomatidae in *Fishes of the north-eastern Atlantic and the Mediterranean* (eds. Whitehead, P.J.P., Bauchot, M.-L., Hureau, J.-C., Nielsen, J. & Tortonese, E.) Vol. 1 284-301 (UNESCO, 1984).

6. Bernal, A., Olivar, M. P., Maynou, F. & Fernández de Puelles, M. L. Diet and feeding strategies of mesopelagic fishes in the western Mediterranean. *Prog. Oceanogr.* **135**, 1-17 (2015).

7. Thompson, S., & Kenchington, T. Distribution and diet of *Cyclothone microdon* (Gonostomatidae) in a submarine canyon. J. Mar. Biol. Assoc. U.K. **97**, 1573-1580 (2017).

8. Maul, G.E., 1986. Melamphaidae in *Fishes of the north-eastern Atlantic and the Mediterranean* (eds. Whitehead, P.J.P., Bauchot, M.-L., Hureau, J.-C., Nielsen, J. & Tortonese, E.) Vol. 2 756-765 (UNESCO, 1986).

9. Gartner, J.V. & Musick, J.A. Feeding habits of the deep-sea fish, *Scopelogadus beanii* (Pisces: Melamphaide), in the western North Atlantic. *Deep Sea Res.* **36**, 1457-1469 (1989).

10. Hulley, P.A. & Paxton, J.R. Myctophidae in *Bony fishes, part 1 (Elopiformes-Scorpaeniformes), The Living Marine Resources of the Eastern Central Atlantic* (eds. Carpenter, K. & De Angelis, N.) Vol. 2 1860–1928 (FAO, 2016).

11. Badcock, J. Sternoptychidae in *Fishes of the north-eastern Atlantic and the Mediterranean* (eds. Whitehead, P.J.P., Bauchot, M.-L., Hureau, J.-C., Nielsen, J. & Tortonese, E.) Vol. 1 302-317 (UNESCO, 1984).

12. Gibbs, R.H.Jr. Chauliodontidae in *Fishes of the north-eastern Atlantic and the Mediterranean* (eds. Whitehead, P.J.P., Bauchot, M.-L., Hureau, J.-C., Nielsen, J. & Tortonese, E.) Vol. 1. 336-337 (UNESCO, 1984).

13. Merrett, N.R. & Roe, H.S.J. Patterns and selectivity in the feeding of certain mesopelagic fishes. *Mar. Biol.* **28**, 115-126 (1974).

14. Sutton, T.T., Hopkins, T.L. Trophic ecology of the stomiid (Pisces: Stomiidae) fish assemblage of the eastern Gulf of Mexico: strategies, selectivity and impact of a top mesopelagic predator group. *Mar. Biol*. **127**, 179-192 (1996).

15. Scott, W.B. & Scott, M.G. Atlantic fishes of Canada. *Can. Bull. Fish. Aquat. Sci*. **219**, 1-731 (1988).

16. Schlitzer, R. *Ocean Data View*, https://odv.awi.de (2018).
